# Dr. Henry A. Tingley

**Published:** 1905-12

**Authors:** 


### OBITUARY NOTES.

---

Dr. Henry A. Tingley, of Susquehana Pa., died at his home, October 28, 1905, aged 85 years. He graduated in medicine at the University of Buffalo in 1848, served as a medical officer in the volunteer army during the Civil War, has been president of the school board of Susquehana, and was a prominent physician for many years in the community in which he lived..

